# Work-Family Conflict of Emergency Nurses and Its Related Factors: A National Cross-Sectional Survey in China

**DOI:** 10.3389/fpubh.2021.736625

**Published:** 2021-10-14

**Authors:** Yafei Wu, Xuan Zhou, Yanhong Gong, Nan Jiang, Mengge Tian, Jiali Zhang, Xiaoxv Yin, Chuanzhu Lv

**Affiliations:** ^1^Department of Social Medicine and Health Management, School of Public Health, Tongji Medical College, Huazhong University of Science and Technology, Wuhan, China; ^2^Department of Anesthesiology, Tongji Hospital, Tongji Medical College, Huazhong University of Science and Technology, Wuhan, China; ^3^Department of Emergency Medicine, Sichuan Provincial People's Hospital, University of Electronic Science and Technology of China, Chengdu, China; ^4^Research Unit of Island Emergency Medicine, Chinese Academy of Medical Sciences (No. 2019RU013), Hainan Medical University, Haikou, China; ^5^Key Laboratory of Emergency and Trauma of Ministry of Education, Hainan Medical University, Haikou, China

**Keywords:** work-family conflict, nurse, emergency department, night shift, cross-sectional study

## Abstract

The prevalence of work-family conflict (WFC) among nurses was high, especially in the emergency department. WFC has a series of negative influences on emergency nurses, but factors associated with WFC require elucidation. Thus, we conducted a national cross-sectional survey among emergency nurses in China. In this study, we described the current situation of WFC and explored its related factors among emergency nurses in China. We found that the WFC of emergency nurses was severe, and emergency nurses aged 25 to 34, male, married, highly educated, with high professional title and long years of service, perceiving the shortage of nurses, experiencing a high frequency of night shift, tended to have higher WFC. Targeted interventions, such as reasonable work allocation, adequate staffing, and a scientific night shift system should be implemented to alleviate the WFC of emergency nurses.

## Introduction

Work-family conflict (WFC) occurs when work or family demands make it hard to fulfill demands in the alternative role (1). Nurses have an increasing workload with the growth of medical demands and irregular working time, which lead to the challenge of balancing work and family (2, 3). Previous studies in many countries confirmed that the prevalence of WFC among nurses was high and mainly at a high level (4–7). As a consequence of the fast work pace, high pressure, and frequent violence in the emergency department (8, 9), the WFC of emergency nurses is more severe than that of nurses in other departments (10).

Work-family conflict has a series of negative impacts on the physical and mental health, work status, and family life of healthcare workers. First of all, WFC can lead to some physical and mental health problems, such as emotional exhaustion, work-related fatigue, sleep disorders, anxiety, and depression (11–14). In addition, WFC is likely to reduce job performance, job satisfaction, and organizational commitment (15, 16) while increase job burnout and turnover intention (17–19), which negatively affect work status and organizational development. Furthermore, pressure arising from WFC might be somewhat transmitted to other family members, resulting in the decline of marital satisfaction and life satisfaction (20, 21). Consequently, we should pay attention to the current situation and relevant factors of WFC among emergency nurses and accordingly devise effective interventions.

At present, studies on the WFC of nurses mainly focused on the situation and its negative influence, but the influencing factors were not paid enough attention. A limited number of previous studies showed that the WFC of nurses was associated with age, working hours, organization factors, and family factors (3, 18, 22). However, the population of those studies was general nurses, and rarely aiming at emergency nurses. Therefore, we carried out a national survey among emergency nurses in China to describe the current situation of WFC and explored its influencing factors. Understanding these factors might provide ideas for interventions to reduce the WFC of emergency nurses, thereby diminish its negative effects such as the increase of the turnover intention, which will finally promote the stability and development of the emergency nursing team.

## Methods

### Participants and Data Collection

This study is part of a nationwide cross-sectional survey of emergency medical resources, conducted with the coordination of the Medical Administration Bureau of the National Health Commission of the People's Republic of China. Data in this study were collected through the online survey platform Questionnaire Star in China. From July to August in 2018, the link of the questionnaire was posted on the working platform for emergency nurses in the pre-hospital emergency facility configuration monitoring department, inviting all emergency nurses to participate anonymously in this online survey. If the questionnaire is incomplete, it cannot be submitted; thus, there is no missing data in each variable. The survey link was resent to the online working platform every 7 days to remind emergency nurses to respond until the end of the survey. All participants were required to read and agree to the electronic Informed Consent Statement before responding. During the study period, a total of 25,518 emergency nurses clicked on the survey link, and 17,582 emergency nurses from 2,965 public hospitals providing pre-hospital care in 31 provinces of China completed the online questionnaire, the response rate was 68.9%.

### Measures

The electronic questionnaires were developed based on a review of published literature. Data were collected using a standard structured anonymous questionnaire administered by an online survey platform in China (platform name: Questionnaire Star, website: https://www.wjx.cn). There were 14 items in the questionnaire, covered socio-demographic characteristics, work-related factors, and WFC. Specifically, four items of socio-demographic characteristics covered age, gender, marital status, and educational level. And five items of work-related factors included professional title, monthly income, years of service, frequency of night shift, and a perceived shortage of nurses. The perceived shortage of nurses was assessed by one question: “Do you think the current number of nurses in the emergency department meets the needs of daily work?” (“Does not meet demands” and “Meets demands”).

The WFC of nurses was assessed by The Work-Family Conflict Scale developed by Netemeyer et al. (23). The scale contains five items, using Likert's 5-level scoring method, ranging from 1 (strongly disagree) to 5 (strongly agree), with a total score of 5 to 25. A higher score reflected a higher level of WFC. According to the standard developed by Estryn et al. (12), the average scores of the five items were divided into three levels: scores less than 2.5 indicated low WFC, scores from 2.5 to 3.6 indicated moderate WFC, and scores greater than or equal to 3.6 indicated high WFC. Previous studies in various Chinese occupational populations have demonstrated satisfactory reliability and validity of the scale (24–27). In this study, the Cronbach's α for the scale was 0.92, indicating good reliability.

### Ethical Considerations

This study was approved by the Research Ethics Committee in Hainan Medical University, Hainan, China (approval number: HYLL-2018-035). Informed consent was obtained from every nurse involved in the investigation. Each participant was assigned a unique code rather than their actual names to ensure confidentiality. All participants were assured that their personal information would be kept confidential.

### Data Analysis

All data were analyzed using IBM SPSS Statistics R23.0 (NY, USA). In descriptive statistics, categorical variables were represented by frequency and percentage, and continuous variables were represented by means and SD. The WFC scores of nurses in different groups were compared by Student's *t*-test or ANOVA, and a separate variance estimation *t*-test and the Welch test were conducted when the homogeneity test of variances was significant. The contingency coefficient in correlation analysis and the variance inflation factor were used to test for multicollinearity between independent variables. The contingency coefficient was below the traditional maximum threshold of 0.7, and the variance inflation factor was below the traditional cutoff value of 10, indicating that no collinearity was detected. And the related factors of WFC of nurses in the emergency department were analyzed by generalized linear regression, as the WFC score was not normally distributed. All comparisons were two-tailed, and the significance threshold was *p*-values < 0.05.

## Results

### General Characteristics of the Participants

The socio-demographic characteristics and working status of the respondents are shown in Table 1. There is no missing data in each variable due to the online survey platform. Among the 17,582 emergency nurses investigated, 10.25% were male and 89.75% were female. Nearly 80% of the emergency nurses were younger than 35 years old, and 61.81% of respondents were married. The educational level was mainly associate degree, accounting for 48.93%. In addition, 66.62% of respondents had the primary professional title, and the monthly income of emergency nurses was mainly 2,500–4,000 RMB (43.13%). And the years of service of them was mainly 1–5 years, accounting for 44.27%. Furthermore, their frequency of night shift was mainly 6–10 days/month (43.90%). And 50.24% of respondents perceived that the number of emergency nurses could not meet the work demands.

**Table 1:** Univariate analysis of work-family conflict of emergency nurses (*n* = 17,582).

**Variables**	**Number, percentage (*N*, %)**	**WFC scores (mean, SD)**	**Statistical values (*F*/*t*)**
**Total**	17,582 (100)	17.55 (4.01)	
**Socio-demographic variables**		
**Age**			113.93^*^
< 25	3,493 (19.87)	16.65 (4.08)	
25–34	10,540 (59.95)	17.85 (4.02)	
>34	3,549 (20.19)	17.57 (3.76)	
**Gender**			6.10^*^
Male	1,803 (10.25)	18.13 (4.26)	
Female	15,779 (89.75)	17.49 (3.97)	
**Educational level^#^**			60.94^*^
Vocational diploma or below	1,105 (6.28)	16.79 (3.87)	
Associate degree	8,603 (48.93)	17.34 (4.03)	
Bachelor degree or higher	7,874 (44.78)	17.89 (3.97)	
**Marital status**			−10.62^*^
Unmarried/other	6,714 (38.19)	17.14 (4.13)	
Married	10,868 (61.81)	17.81 (3.91)	
**Work-related variables**
**Title**			43.15^*^
None	2,328 (13.24)	16.88 (4.14)	
Primary	11,713 (66.62)	17.60 (4.02)	
Medium or higher	3,541 (20.14)	17.86 (3.83)	
**Monthly income (RMB)**			9.66^*^
≤ 2499	2,980 (16.95)	17.24 (4.18)	
2500–4000	7,583 (43.13)	17.53 (4.01)	
4001–6000	4,649 (26.44)	17.71 (3.88)	
≥6001	2,370 (13.48)	17.73 (3.99)	
**Years of service**			91.53^*^
< 1	2,540 (14.45)	16.48 (4.04)	
1–5	7,783 (44.27)	17.51 (4.01)	
6–10	4,573 (26.01)	18.08 (3.97)	
≥11	2,686 (15.28)	17.83 (3.82)	
**Frequency of night shift (per month)**			145.64^*^
0–5	5,417 (30.81)	16.90 (3.88)	
6–10	7,719 (43.90)	17.45 (3.95)	
11–15	3,651 (20.77)	18.35 (3.99)	
≥16	795 (4.52)	19.36 (4.35)	
**Perceived shortage of nurses**			−39.79^*^
No	8,748 (49.76)	16.40(3.83)	
Yes	8,834 (50.24)	18.70 (3.84)	

*WFC, work-family conflict*.

# *A vocational diploma requires 2 years of education in vocational schools after graduation from senior middle school or 3 years of education in vocational schools after graduation from junior middle school, and an associate degree requires 3 years of education in college after graduation from senior middle school (grade year 10 to year 12), or 5 years of education in college after graduation from junior middle school (grade year 7 to year 9)*.

* *Correlation is significant at the 0.001 level (two-tailed)*.

### Work-Family Conflict of Emergency Nurses

Table 1 shows the diversity of WFC among emergency nurses with different characteristics. The average score of WFC of respondents was 17.55 (SD = 4.01), and the average score of each item was 3.51 (SD = 0.80). Specifically, 9.99% of emergency nurses presented low WFC, 47.30% presented moderate WFC, and 42.71% presented high WFC. Additionally, the univariate analysis demonstrated that there were significant differences in WFC among emergency nurses in terms of age, gender, educational level, marital status, professional title, monthly income, years of service, frequency of night shift, and a perceived shortage of nurses.

### Generalized Linear Regression Analysis

Generalized linear regression was conducted to examine factors associated with the WFC of emergency nurses. As shown in Table 2, the WFC scores of emergency nurses aged 25–34 years were higher than that of nurses under 25 years old (*p* = 0.001), and the emergency nurses who were male (*p* < 0.001) or married (*p* < 0.001) presented higher WFC scores. Compared with emergency nurses with the vocational diploma or below, nurses who attended associate degree (*p* = 0.015) and bachelor degree or higher (*p* < 0.001) showed higher WFC scores. In terms of work-related factors, nurses with the primary professional title (*p* = 0.017), medium or higher professional title (*p* < 0.001), long years of service (*p* < 0.001), and high frequency of night shift per month (*p* < 0.001) presented higher WFC scores. In addition, emergency nurses who perceived the shortage of nurses scored higher in WFC (*p* < 0.001).

**Table 2:** Generalized linear regression examining factors associated with work-family conflict.

	**β**	**SE**	***t***	***p-*value**
**Socio-demographic variables**				
Age (Ref^*^:< **25)**
25–34	0.30	0.09	3.29	0.001
>34	−0.24	0.13	−1.79	0.074
**Gender (Ref** = **Male)**				
Female	−0.56	0.10	−5.82	< 0.001
**Educational level (Ref** = **Vocational diploma or below)**				
Associate degree	0.30	0.12	2.45	0.015
Bachelor degree or higher	0.49	0.13	3.87	< 0.001
**Marital status (Ref** = **Unmarried/other)**				
Married	0.29	0.07	4.09	< 0.001
**Work-related variables**				
**Title (Ref** = **None)**				
Primary	0.21	0.09	2.39	0.017
Medium or higher	0.44	0.13	3.51	< 0.001
**Monthly income (RMB) (Ref:** ≤ **2,499)**				
2,500–4,000	−0.04	0.08	−0.45	0.653
4,001–6,000	−0.04	0.09	−0.45	0.651
≥6001	−0.08	0.11	−0.69	0.490
**Years of service (Ref:**< **1)**				
1–5	0.58	0.09	6.41	< 0.001
6–10	0.84	0.11	7.83	< 0.001
≥11	0.89	0.13	6.79	< 0.001
**Frequency of night shift (per month) (Ref** = **0**–**5)**				
6–10	0.31	0.07	4.63	< 0.001
11–15	1.02	0.08	12.36	< 0.001
≥16	1.89	0.15	13.03	< 0.001
**Perceived shortage of nurses (Ref** = **No)**				
Yes	1.35	0.04	36.60	< 0.001

*F = 129.79, p < 0.001. R^2^ was 0.117, and the adjusted R^2^ was 0.117, indicating that approximately 11.7% of WFC scores could be explained by the model*.

* *Ref is a reference*.

## Discussion

This study described the current situation of WFC among emergency nurses in China and explored its relationship with socio-demographic characteristics and work-related factors. We found that the average WFC score of emergency nurses in China (3.51) was higher than that reported in other countries, including developed countries such as France (2.9), Germany (2.8) (28), and Italy (2.84) (29) and developing countries such as Ghana (3.28)(2), Croatia (3.43) (30), and Iran (2.74) (31), whereas it is difficult to make direct comparison due to the changes in the study population. Moreover, nearly half of the emergency nurses were faced with high WFC, indicating that the WFC of emergency nurses in China was particularly serious and should be paid more attention to.

The results of linear regression exhibited that the WFC of emergency nurses aged 25–35 and married was especially pronounced. The age of 25 to 35 might be the golden period for the career development of emergency nurses, who usually undertook quantities of complicated nursing tasks (32). Meanwhile, it was also a period when marriage and family were just established and unstable yet, crucial problems such as maintaining family and parenting emerged (20, 33). Consequently, emergency nurses of this age group and those who are married have more difficulties in achieving work-family balance and are more prone to WFC. In addition, male nurses in the emergency department had higher WFC, which might be associated with the fact that male nurses often undertook high physical consumption and challenging work in clinical nursing (34). However, the gender difference in WFC between different countries was inconsistent (3, 35) due to the distinctions in the social system, culture, and traditional concepts in different countries (36). Furthermore, highly educated emergency nurses showed higher WFC, which might be related to the fact that emergency nurses with higher education tended to undertake more complex tasks and heavier responsibility in the nursing team (37). Therefore, nursing managers should pay close attention to emergency nurses aged 25 to 35, married, associate degree or above, and ensure reasonable work allocation of them.

Work-related factors were strongly associated with the WFC of emergency nurses. This study found that WFC tended to be more serious in emergency nurses with a high professional title and long years of service. Those nurses undoubtedly possessed more adequate professional knowledge and richer work experience and were the backbone of emergency nursing work. The conflict is that majority of them simultaneously had increasing family role demands of maintaining daily life or raising children (38). Therefore, we should pay attention to emergency nurses with high professional titles and long years of service in particular and reasonably allocate the nursing work. In addition, overwork and irregular working time were significant predicting factors of WFC (28, 39). According to our study, perceived shortage of nurses in the emergency department and high frequency of night shift were significantly correlated with high WFC. It might be speculated that when the number of nurses could not meet the work demands in an emergency department, the work intensity of nurses on staff might increase, implying higher workloads (3). Moreover, a high frequency of night shifts also led to increased workload (33), and irregular working time might lead to sleep disorders simultaneously (28, 40), thereby possibly had an impact on private life. Accordingly, it is necessary to ensure adequate emergency nurses (3) and reasonably adjust working time and night shift frequency (10, 41).

In order to promote the sustainable development of the emergency nursing team, it is important for hospital administrators and policymakers to develop policy strategies and take actions to mitigate WFC among emergency nurses. Such strategies should include adequate staffing to ensure that sufficient nurses are available to care for patients, meanwhile, reduce the risk of overwork for each nurse. This should also include rational allocation of nursing work and adopting more efficient clinical nursing models to maximize nursing efficiency. In addition, reasonable shift systems are essential to reduce the WFC of emergency nurses. Appropriate night shift frequency enables emergency nurses to meet the needs of nursing work while their family life will not be seriously disturbed. Finally, we should improve the vacation system, especially promote the implementation of marriage leave and maternity leave, so that emergency nurses can better maintain the balance between family and work in the particular stage.

This study was the first national cross-sectional survey of WFC among emergency nurses in China. We described the current situation of WFC among emergency nurses in China, identified relevant factors, and provided valuable references for the formulation of interventions. The sample in this study was representative owing to the large sample size and nationwide survey. However, there are some limitations to our study. First, the cross-sectional design of this study cannot draw a clear causal conclusion, and further prospective research is needed. Second, all participants were from public hospitals, which may diminish the representativeness of samples. Third, our study was conducted in China, the findings should be carefully generalized in other countries. Finally, convenience sampling and online surveys were adopted in this study; thus, the majority of respondents were young people who used the Internet, which might lead to the preferences of the participants.

## Conclusions

The current situation of WFC among emergency nurses in China was serious, especially for the emergency nurses aged 25 to 35, male, married, highly educated, with high professional title and long years of service. Targeted measures should be adopted to improve WFC. On the one hand, we should reduce the workload and work pressure of nurses in the emergency department through reasonable work allocation and adequate staffing. On the other hand, a scientific shift system should be established to enhance the working time flexibility of emergency nurses, so as to improve the combination of their work with family life.

## Data Availability Statement

The datasets presented in this article are not readily available because the data also forms part of an ongoing study. Requests to access the datasets should be directed to Xiaoxv Yin, yxx@hust.edu.cn.

## Ethics Statement

The studies involving human participants were reviewed and approved by the Research Ethics Committee in Hainan Medical University, Hainan, China (Approval Number: HYLL-2018-035). The patients/participants provided their written informed consent to participate in this study.

## Author Contributions

YW, XZ, YG, and XY contributed to the study design. CL and XY were involved in the data collection. YW, MT, and JZ performed the data analysis. XY, CL, and NJ supervised the study. YW, XZ, and YG contributed to the manuscript writing. YW, NJ, and XY involved in critical revisions for important intellectual content. All authors contributed to the article and approved the submitted version.

## Funding

This study was funded by the Department of Science and Technology of Hainan Province, Major Science and Technology Projects (ZDKJ202004) and Department of Science and Technology of Hainan Province, Key Research and Development Program (ZDYF2020112).

## Conflict of Interest

The authors declare that the research was conducted in the absence of any commercial or financial relationships that could be construed as a potential conflict of interest.

## Publisher's Note

All claims expressed in this article are solely those of the authors and do not necessarily represent those of their affiliated organizations, or those of the publisher, the editors and the reviewers. Any product that may be evaluated in this article, or claim that may be made by its manufacturer, is not guaranteed or endorsed by the publisher.
